# Longitudinal imaging of murine atherosclerosis with 2-deoxy-2-[^18^F]fluoro-D-glucose and [^18^F]-sodium fluoride in genetically modified Apolipoprotein E knock-out and wild type mice

**DOI:** 10.1038/s41598-023-49585-1

**Published:** 2023-12-27

**Authors:** Harshvardhan A. Khare, Tina Binderup, Anne Mette Fisker Hag, Andreas Kjaer

**Affiliations:** Department of Clinical Physiology and Nuclear Medicine & Cluster for Molecular Imaging, Copenhagen University Hospital - Rigshospitalet & Department of Biomedical Sciences, University of Copenhagen, Copenhagen, Denmark

**Keywords:** Cardiology, Cardiovascular biology, Calcification, Experimental models of disease

## Abstract

In a longitudinal design, four arterial segments in mice were followed by positron emission tomography/computed tomography (PET/CT) imaging. We aimed to determine how the tracers reflected the development of atherosclerosis via the uptake of 2-deoxy-2-[^18^F]fluoro-D-glucose ([^18^F]FDG) for imaging inflammation and [^18^F]-sodium fluoride (Na[^18^F]F) for imaging active microcalcification in a murine model of atherosclerosis. Apolipoprotein E knock-out (ApoE) mice and C57 BL/6NtaC (B6) mice were divided into four groups. They received either normal chow (N = 7, ApoE mice and N = 6, B6 mice) for 32 weeks or a high-fat diet (N = 6, ApoEHFD mice and N = 9, B6HFD mice) for 32 weeks. The mice were scanned with [^18^F]FDG and Na[^18^F]F using a dedicated small animal PET/CT scanner at three timepoints. The tracer uptakes in four aortic segments (abdominal aorta, aortic arch, ascending aorta, and thoracic aorta) were measured and reported as SUV_max_ values. The uptake of [^18^F]FDG (SUV_max_: 5.7 ± 0.5 vs 1.9 ± 0.2, 230.3%, p =  < 0.0001) and Na[^18^F]F (SUV_max_: 9.6 ± 1.8 vs 4.0 ± 0.3, 175%, p = 0.007) was significantly increased in the abdominal aorta of ApoEHFD mice at Week 32 compared to baseline abdominal aorta values of ApoEHFD mice. [^18^F]FDG uptake in the aortic arch, ascending aorta and the thoracic aorta of B6HFD mice at Week 32 showed a robust resemblance to the abdominal aorta uptake whereas the Na[^18^F]F uptake only resembled in the thoracic aorta of B6HFD mice at Week 32 compared to the abdominal aorta. The uptake of both [^18^F]FDG and Na[^18^F]F increased as the disease progressed over time, and the abdominal aorta provided a robust measure across mouse strain and diet. Therefore, it seems to be the preferred region for image readout. For [^18^F]FDG-PET, both B6 and ApoE mice provide valuable information and either mouse strain may be used in preclinical cardiovascular studies, whereas for Na[^18^F]F -PET, ApoE mice should be preferred.

## Introduction

Atherosclerosis is associated with age, gender, smoking, obesity, and diet, amongst other risk factors^1–4^. The disease progresses over time and often results in inflammation and mineralization in the vessel wall leading to the formation of atherosclerotic plaques. Identification of regions with increased disease activity and consequently vulnerable atherosclerotic plaques, may be a predictor of subsequent adverse events^5,6^.

Using hybrid positron emission tomography and computed tomography (PET/CT), it is possible to track the progression of key molecular markers non-invasively with a high level of sensitivity and accuracy^7–9^. 2-deoxy-2-[^18^F]fluoro-D-glucose ([^18^F]FDG) PET imaging has previously been studied in humans and in animals for visualization of vascular inflammation, a key feature in atherogenesis^10–15^. Another PET tracer, ^18^F-sodium fluoride (Na[^18^F]F), has the ability to detect localized spotty calcifications, microcalcifications and active calcifications; a vital feature of atherogenesis that occurs in progressive necrotic areas and its use in imaging of atherosclerosis has recently been reconsidered^16–19^. Na[^18^F]F imaging provides a different dimension to visualization of calcification as compared to CT^20^. A previous study of the abdominal aorta suggested that Na[^18^F]F uptake does not change despite an increase in CT detectable-calcification^21^.

A commonly used mouse model of atherosclerosis is the Apolipoprotein E knock-out (ApoE) mouse fed a high fat diet (HFD). However, the ApoE mouse model is costly and although the general belief is that normal, wild type mice are not suitable for the study of atherogenesis and atherosclerosis, this has not been substantiated in the literature. Therefore, in the present study we performed longitudinal imaging experiments of atherosclerotic development in both ApoE and wild type C57 BL/6NtaC (B6) mice.

The aim of this study was to evaluate and directly compare the uptake of [^18^F]FDG and Na[^18^F]F in a longitudinal, paired study. This allowed for the study of disease progression via molecular changes in inflammation and mineralization. Tracer uptake was quantified in four aortic segments, namely the suprarenal abdominal aorta, descending thoracic aorta, the ascending aorta, and the aortic arch. In addition, we obtained data on whether a wild type mouse strain B6 can be used for studies of atherogenesis or it is necessary to use the genetically modified ApoE mouse.

## Materials and methods

### Ethical statement

All animal experiments were approved and were performed in compliance to the Animal Experiments Inspectorate in Denmark, Danish Ministry of Justice (License no.: 2007–561-1328) as well as the ARRIVE, AVMA guidelines and regulations, severity protocols. The study was approved by Department of Experimental Medicine, University of Copenhagen.

### Experimental model

Homozygous male apoE^−/−^ mice (B6.129P2) and C57 BL/6NtaC (B6 mice) mice, 7 weeks old, were purchased from Taconic (Taconic Europe, Denmark). The mice were fed a normal chow diet (NCD) until the initiation of experimental diets.

Throughout the course of experiments, the mice were housed under controlled humidity, temperature, and light cycle conditions, and had free access to food and water.

**Figure 1:**
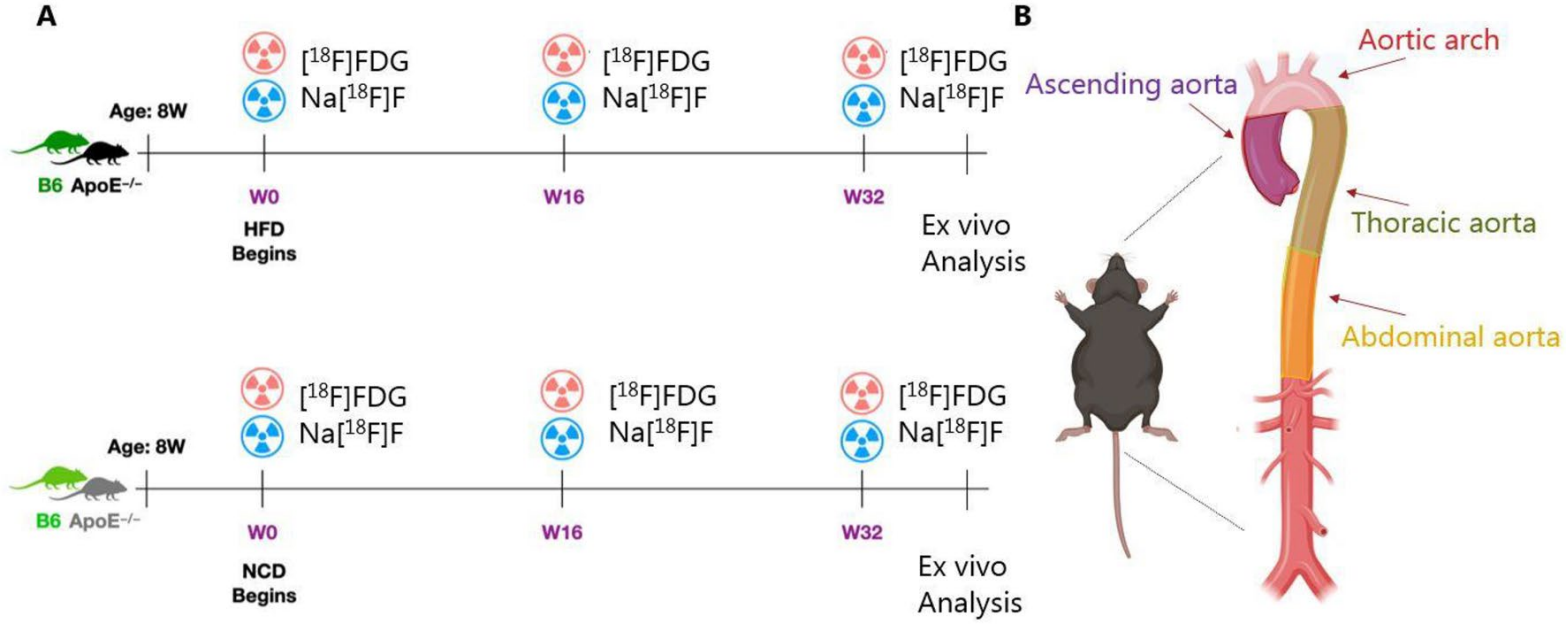
(**A**) Schematic representation of experimental setup. (**B**) An illustration of segmentation of mouse aorta into four segments namely the ascending aorta, the aortic arch, the thoracic aorta and the abdominal aorta.

The mice were divided into four groups as follows: Group 1 (B6 mice, n = 7, called B6 henceforth) and Group 3 (apoE^−/−^ mice, n = 9, called ApoE henceforth) received normal chow diet (NCD) for 32 weeks whereas Group 2 (B6 mice, n = 6, called B6HFD henceforth) and Group 4 (apoE^−/−^ mice, n = 7, called ApoEHFD henceforth) received a high-fat Western type diet (HFD) for 32 weeks. The high fat Western type diet contained 21% fat and 0.21% cholesterol (Diet#D12079B, Research Diets, Inc., USA). All mice were sacrificed after the terminal scan at Week 32, whole aortas were removed, gamma counted. Table 1 provides an overview of animals and diets. A graphical representation of the study is illustrated in Fig. 1A.

**Table 1:** Overview of number of animals used per group and their diet regime.

Group 1–4	No. of animals	Weeks on HFD	Weeks on NCD
B6	7	0	32
B6HFD	6	32	0
ApoE	9	0	32
ApoEHFD	7	32	0

Some mice died during the course of 32 weeks and between endpoint scans of the two tracers and resulted in reduction of number of mice in imaging time points. A minimum of n = 5 animals remained per group at every imaging time point.

### Experimental protocol

All the mice were scanned with both [^18^F]FDG & Na[^18^F]F at baseline (Week 0, W0, at the initiation of diet, at Week 16 (W16) , and a terminal scan at Week 32 (W32). Refer to Fig. 1A for a schematic representation of the experimental setup. Prior to PET/CT scans, all animals were fasted overnight. All animals were gas anaesthetized with a mixture of 3% sevoflurane (Abbot Scandanavia AB, Sweden), 35% O_2_ in N_2_. The animals were rested at approximately 32 °C post injection and kept warm throughout the PET/CT scans.

[^18^F]FDG and Na[^18^F]F was produced and obtained from Department of Clinical Physiology, Nuclear Medicine and PET, Rigshospitalet, Copenhagen, Denmark. 20.1 ± 4.8 MBq of [^18^F]FDG in saline and 11.9 ± 1.6 MBq of Na[^18^F]F in saline was administered i.v in a lateral tail vein with a vein catheter (BD Vasculon™ Plus, Becton Dickinson A/S, Denmark). A long circulating CT contrast (Fenestra VC^®^, MediLumine Inc., Montreal, QC, Canada) at a concentration of 0.2 ml/20 g was administered through the same vein catheter following the injection of PET tracer. A 30-min PET scan was carried out 3 h post injection for [^18^F]FDG and 60 min post injection for Na[^18^F]F, followed immediately by a CT scan. It has been previously shown that more than 120 min post injection is the optimal time point for [^18^F]FDG imaging of vessel walls to minimize background accumulation^11,22^. Both scans were carried out with the animal in prone position and in the same acquisition bed to maintain exact position in both scans (PET and CT).

### PET protocol

PET scans were carried out with a microPET Focus 120 scanner (Siemens Medical Solutions, Erlangen, Germany). Emission data was normalized, decay and dead time corrected. Listmode data were saved automatically post scan and processed into 128 × 144 × 95 sinograms with a ring difference of 47 and span of three. A 3D Maximum a posteriori (MAP) algorithm was applied to sinograms for reconstruction^23^. Resolution was set to 1.2 mm full-width at half-maximum and voxel size was 0.3 × 0.3 × 0.79 mm^3^.

### CT protocol

CT scans were carried out with a MicroCAT II scanner (Siemens Medical Solutions, USA). The X-ray tube current was set to 500µA with a 0.5 mm aluminium filter at 60 kVp and an exposure time of 310 ms per projection. 360 projections were used to create a full 360̊ scan. A voxel size of 0.095 × 0.095 × 0.095 mm^3^ was used for image reconstruction using COBRA real-time reconstruction with the Sheep-Logan filter.

Inveon Research Workplace Version 4.2 (Siemens Medical Solutions USA, Inc) image analysis software was used to quantify [^18^F]FDG and Na[^18^F]F uptake in the aorta of each animal. PET and CT scans were fused by using the rigid registration function, followed by manual visual confirmation. The confirmation was based on locating renal artery that connects the abdominal aorta. Refer to Supplementary Figs. 1, 2 and 3 for examples of segmentation of aortas, ROI volume construction in the supplementary data section.

### PET quantification

Four anatomical territories were chosen for analysis: The suprarenal abdominal aorta (cranially from the renal bifurcature approximately 30 slices), the thoracic aorta (cranially from the top slice of abdominal aorta until the beginning of the aortic arch, approximately 30 slices), and the ascending aorta (cranially from the root of ascending aorta until beginning of the aortic arch, approximately 5 slices) and the aortic arch (cranially from the top of ascending aorta until the top slice of thoracic aorta). Regions of interest (ROIs) were drawn on CT scans (superimposed on PET scans) by free hand to include vessel lumen and vessel wall in all vascular territories. Volumes of interest (VOIs) were manually constructed based on ROIs on specific aorta sections. Refer to Fig. 1B for an illustration of aortic segments considered for analysis. Maximum standardized uptake values (SUV_max_) that are highest image pixels in the ROIs were calculated with the following equation:

$${\text{SUV}}_{{{\text{max}}}} = \, \frac{{{\text{Maximum radioactivity concentration in regions of interest }}\left( {{\text{MBq}}/{\text{mL}}} \right)}}{{\frac{{{\text{Injected dose }}\left( {{\text{MBq}}} \right)}}{{{\text{Weight of animal }}\left( {\text{g}} \right)}}}}$$

The average of SUV_max_ from each aortic segment was considered for quantification.

ROIs were drawn on muscle (Tibialis Anterior, TA) and brown adipose tissue (BAT,neck region) in order to calculate Maximum tumor to background ratios (TBR_max_):

$${\text{TBR}}_{{{\text{max}}}} = \, \frac{{{\text{Maximum radioactivity concentration in regions of interest }}\left( {{\text{Aorta}},{\text{ in MBq}}/{\text{ml}}} \right)}}{{{\text{Mean radioactivity concentration in regions of background }}\left( {{\text{BAT or TA}},{\text{ in MBq}}/{\text{ml}}} \right)}}.$$

The average of TBR_max_ values were considered for quantification.

### Gamma counting

Freshly excised whole aortas post animal sacrifice were gamma counted on a Wizard 2 gamma counter (Perkin Elmer, Massachusetts, USA). 480–558 keV energy window and an acquisition period of 120 s per sample were used for the gamma counting protocol.

### Von Kossa staining

Excised whole aortas along with the aortic root were washed with saline, fixed with 10% formalin solution for 24 h and embedded in paraffin. Paraffin embedded tissue was serially sectioned (10 µm) and to identify vascular calcification, Von Kossa staining was performed. The sections were deparrafinized and rehydrated in water followed by incubation in silver nitrate solution (5%) for 60 min along with exposure to ultraviolet light. The sections were rinsed with distilled water and incubated in sodium thiosulfate solution (5%) for 3 min. The sections were further rinsed under running tap water and mounted on clean glass slides. The slides were scanned with a Zeiss AxioScan.Z1 slide scanner with a Plan Apochromat 10 × objective.

### Statistical analysis

Statistical analysis was conducted using GraphPad Prism 9.0 software (GraphPad Software Inc., USA). Data were presented as mean ± standard error of mean (SEM). A parametric unpaired t test was performed to determine differences between two groups and a *p* value of less than 0.05 was considered statistically significant.

## Results

All the mice were PET/CT scanned longitudinally at Week 0 (Baseline), 16 and 32 (Study termination) with both [^18^F]FDG & Na[^18^F]F. Uptake of [^18^F]FDG and Na[^18^F]F was measured in 4 aortic segments and compared to determine whether changes reflecting development of atherosclerosis are observed in a particular aortic segment, mouse type and time point.

**Figure 2:**
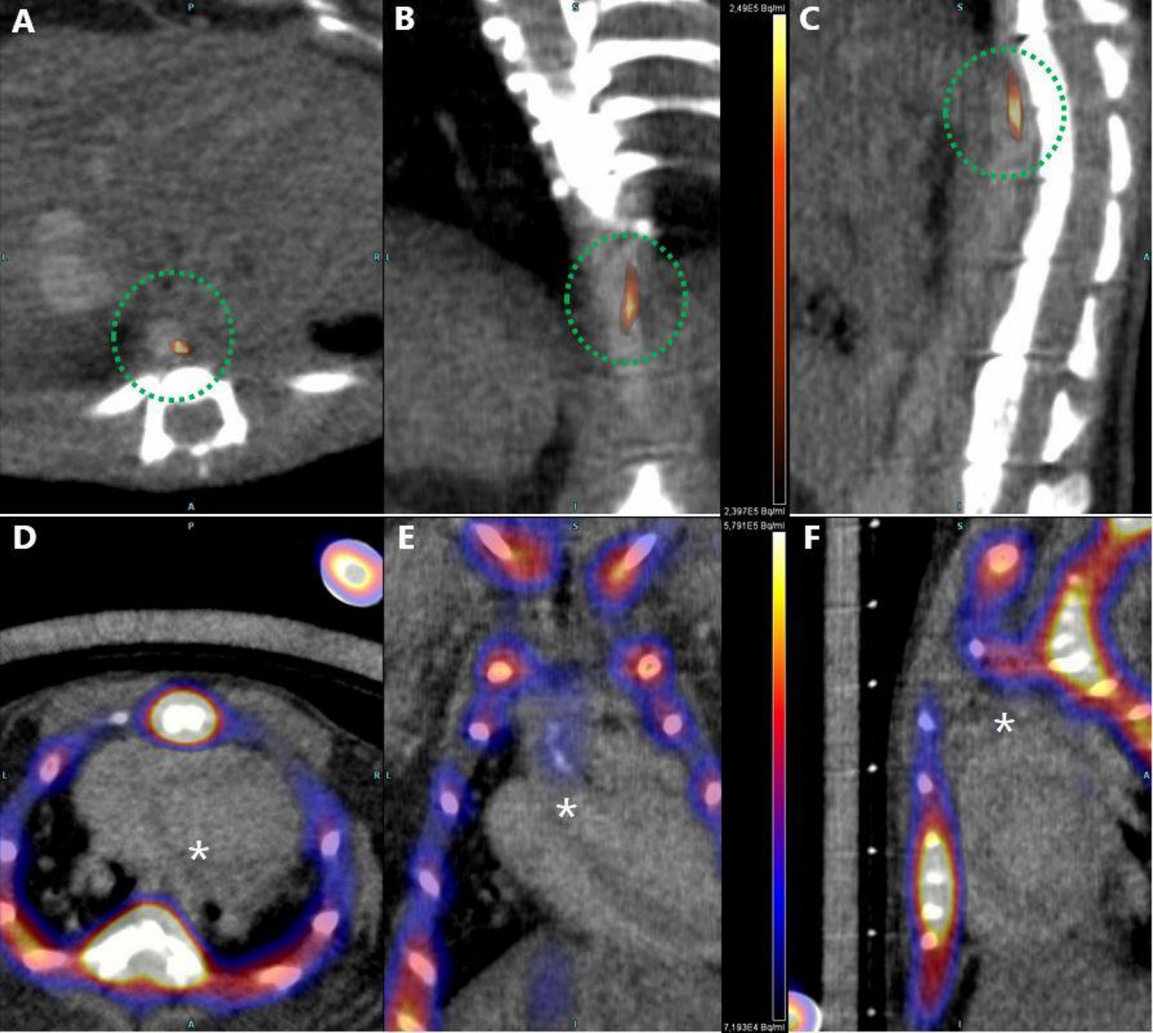
Visualization of inflammation and active microcalcification in representative [^18^F]FDG & Na[^18^F]F PET/CT scans in murine models of atherosclerosis. (**A**–**C**) show accumulation of [^18^F]FDG in the thoracic aorta (highlighted by a green dotted circle) and Na[^18^F]F accumulation in the aortic arch ((**E**), highlighted by an asterisk) along with obvious accumulation in the skeleton (**D**,**F**) of a mouse from ApoEHFD group. Left to right -Axial, Coronal, Sagittal views. *L* Left, *R* Right, *A* Anterior, *P* Posterior.

Representative examples of visualization of inflammation and microcalcification via uptake of [^18^F]FDG and Na[^18^F]F, respectively, are shown in Fig. 2.

### Development of atherosclerosis visualized by [^18^F]FDG PET

**Figure 3:**
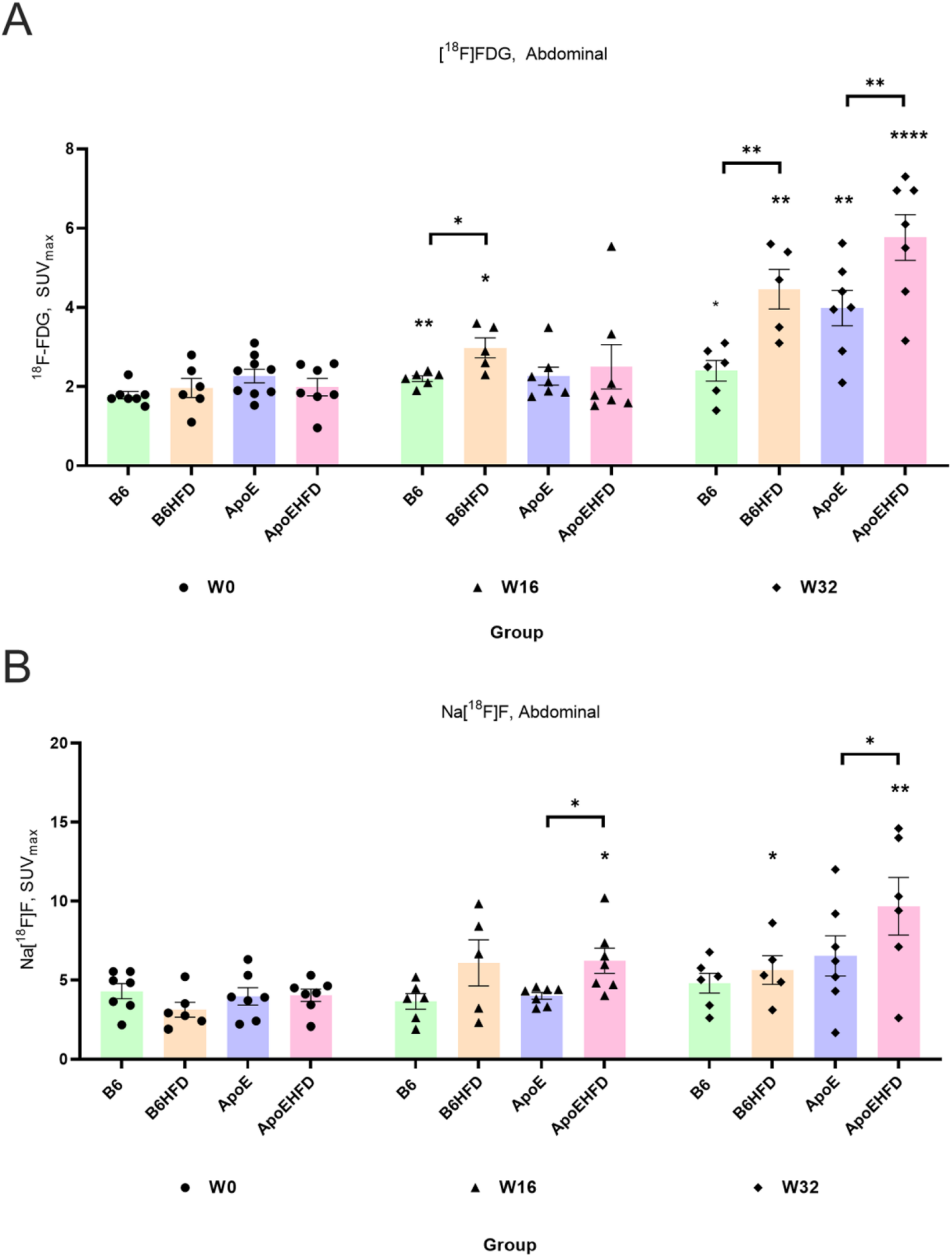
(**A**) [^18^F]FDG and & (**B**) Na[^18^F]F PET/CT imaging of atherosclerosis in abdominal aorta respectively. Highest uptakes and increases in mean SUVmax values of 18[F]FDG and Na[^18^F]F were observed in ApoEHFD mice in the abdominal aorta at W32. SUV_max_ = Maximum of Standardized uptake values with mean ± std. error of mean, [^18^F]FDG = 2-deoxy-2-[^18^F]fluoro-D-glucose. Na[^18^F]F = ^18^F-sodium fluoride. Asterisk represent significant differences compared to baseline average SUV_max_ values from the same mice group. Asterisks on top of a line represent differences between two groups of mice.

We evaluated how ^18^[F]FDG accumulation was affected by diet and mouse strain over time in the abdominal aorta. At 16 weeks following diet initiation, a significant increase was observed in the B6 (SUV_max_: 2.2 ± 0 vs 1.7 ± 0, 23.2%, p = 0.006, Fig. 3A) and the B6HFD (SUV_max_:2.9 ± 0.2 vs 1.9 ± 0.2, 51.5%, p = 0.001, Fig. 3A) groups compared to baseline. ^18^[F]FDG uptake was also increased in the ApoEHFD group by 45% (SUV_max_:2.5 ± 0.5 vs 1.9 ± 0.2, Fig. 3A), although not significant, while no change was observed in the ApoE group at W16 (SUV_max_:2.2 ± 0.2 vs. 2.2 ± 0.1, Fig. 3A). At 32 weeks following diet initiation, all 4 groups had significantly higher ^18^[F]FDG accumulation in the abdominal aorta compared to baseline. This increase was most pronounced in the ApoEHFD group where a 230% increase was observed compared to baseline (SUV_max_:5.7 ± 0.5 vs. 1.9 ± 0.2, p =  < 0.0001, Fig. 3A).

At 16 weeks, the B6 strain seemed most affected by the diet, where the B6HFD group had significantly higher uptake than the B6 group, while no significant difference was found between the ApoE and ApoEHFD groups. Both strains had significantly higher ^18^[F]FDG uptake in the HFD groups at 32 weeks compared to the groups on normal chow diet, (B6 vs. B6HFD, SUV_max_:2.4 ± 0.2 vs. 4.4 ± 0.5, p = 0.01, Fig. 3A), (ApoE vs ApoEHFD, SUV_max_:3.9 ± 0 vs. 5.7 ± 0.5, p = 0.01) (Fig. 3A and Table 2).

**Table 2:** SUV_max_ (mean ± SEM) [^18^F]FDG & Na[^18^F]F uptakes in different mice groups and aortic segments.

Mice	W0 SUV_max_	W16 SUV_max_	W32 SUV_max_	% Change W0 vs W16, p value	% Change W0 vs W32, p value
Thoracic aorta [^18^F]FDG					
B6	1.4 ± 0.1	2.7 ± 0.1	2.1 ± 0.2	83.65, 0.02*	43.26, 0.02*
B6HFD	1.4 ± 0.1	2.7 ± 0.1	4.7 ± 0.3	86.85, 0.0002***	230.79, < 0.0001****
ApoE	1.3 ± 0	1.2 ± 0.1	1.8 ± 0.4	− 9.31, ns	35.34, ns
ApoEHFD	1.6 ± 0.0	1.8 ± 0.5	2.8 ± 0.4	31.32,ns	41.71, 0.01*
Thoracic aorta Na[^18^F]F					
B6	4.4 ± 0.3	4.2 ± 0.5	6.5 ± 0.5	− 6.13, ns	46.39, 0.007**
B6HFD	4.1 ± 0.5	7.1 ± 0.7	5.9 ± 1.1	70.25, 0.008**	42.89, ns
ApoE	6.2 ± 0.4	3.5 ± 0.2	4.6 ± 0.6	− 43.25, 0.0002***	− 26.81, ns
ApoEHFD	5.3 ± 0.3	5.5 ± 0.3	12.4 ± 1.2	12.44, ns	153.79, 0.0001***
Ascending aorta [^18^F]FDG					
B6	0.9 ± 0	1.0 ± 0.1	2.1 ± 0.5	15.76, ns	131.55, 0.04*
B6HFD	1.1 ± 0	1.0 ± 0	3.8 ± 0.5	− 6.13, ns	240.96, 0.000***
ApoE	1.2 ± 0.1	0.9 ± 0.1	1.7 ± 0.2	− 25.51, ns	41.72, ns
ApoEHFD	0.9 ± 0.0	0.7 ± 0.1	3.2 ± 0.1	− 15.83, ns	250, < 0.0001****
Ascending aorta Na[^18^F]F					
B6	0.6 ± 0	1.2 ± 0.5	0.3 ± 0	95.68, ns	− 48.03, 0.009**
B6HFD	0.8 ± 0.2	1.3 ± 0.7	0.8 ± 0	71.92, ns	10.80, ns
ApoE	0.5 ± 0	0.8 ± 0.3	0.5 ± 0.1	65.22, ns	2.87, ns
ApoEHFD	0.5 ± 0.1	0.7 ± 0.0	1.7 ± 0.3	41.80, ns	265.37, 0.005**
Abdominal aorta [^18^F]FDG					
B6	1.7 ± 0	2.2 ± 0	2.4 ± 0.2	23.2, 0.006**	34.4, 0.03*
B6HFD	1.9 ± 0.2	2.9 ± 0.2	4.4 ± 0.5	51.52, 0.01*	126.77, 0.001**
ApoE	2.2 ± 0.1	2.2 ± 0.2	3.9 ± 0.4	− 0.26, ns	75.74, 0.001**
ApoEHFD	1.9 ± 0.2	2.5 ± 0.5	5.7 ± 0.5	45.03, ns	230.35, < 0.0001****
Abdominal aorta Na[^18^F]F					
B6	4.2 ± 0.4	3.6 ± 0.4	4.7 ± 0.6	− 15.04, ns	11.80, ns
B6HFD	3.1 ± 0.4	6.0 ± 1.4	5.6 ± 0.9	95.45, ns	80.99, 0.02*
ApoE	3.9 ± 0.5	3.9 ± 0.2	6.5 ± 1.2	0.79, ns	64.69, ns
ApoEHFD	4.0 ± 0.3	6.2 ± 0.7	9.6 ± 1.8	60.68, 0.02*	175.03, 0.007**
Aortic arch [^18^F]FDG					
B6	1.6 ± 0	2.2 ± 0	2.3 ± 0.2	41.2, 0.03*	25.95, 0.04*
B6HFD	1.5 ± 0.2	2.5 ± 0.4	3.0 ± 0.5	64.13, 0.0002***	100.86, < 0.0001****
ApoE	1.4.0 ± 0.1	1.1 ± 0	0.9 ± 0.4	− 18.4, ns	− 34.19.94, ns
ApoEHFD	1.3 ± 0.0	1.2 ± 0.1	1.5 ± 0.1	− 11.59, ns	12.05, 0.016*
Aortic arch Na[^18^F]F					
B6	2.0 ± 0.2	1.4 ± 0.2	1.4 ± 0.1	− 29.71, ns	− 28.05, ns
B6HFD	2.3 ± 0.5	3.3 ± 0.2	4.4 ± 0.8	− 42.43, ns	87.98, ns
ApoE	1.7 ± 0.2	1.5 ± 0.1	2.2 ± 0.3	− 12.47, ns	26.16, ns
ApoEHFD	1.5 ± 0.2	1.4 ± 0.2	2.2 ± 0.1	− 8.29, ns	43.62, ns

### Development of atherosclerosis visualized by Na[^18^F]F PET

In addition to ^18^[F]FDG, the accumulation of Na[^18^F]F in the abdominal aorta was assessed at the same time points (W0, W16 & W32). While a significant increase was observed in the ApoEHFD group (SUV_max_:6.2 ± 0.7 vs. 4.0 ± 0.3, 60.6%, p = 0.02, Fig. 3B), none of the other groups had significantly elevated Na[^18^F]F uptake at 16 weeks compared to baseline. At 32 weeks, a further increase was observed in the ApoEHFD group compared to baseline (SUV_max_:9.6 ± 1.8 vs. 4.0 ± 0.3, 175%, p = 0.007, Fig. 3B). A smaller but significant increase was also observed in the B6HFD group at 32 weeks compared to baseline (SUV_max_:5.6 ± 0.9 vs. 3.1 ± 0.4, 80.9%, Fig. 2B), while no significant change was seen for any of the groups on normal chow diet.

There were significant differences observed between ApoE and ApoEHFD (SUV_max_:3.9 ± 0.2 vs. 6.2 ± 0.7, p = 0.01, Fig. 2B) at W16 and at W32 (SUV_max_:6.5 ± 1.2 vs. 9.6 ± 1.8, p = 0.01) (Fig. 3B and Table 2).

### Regional aortic visualization of atherosclerosis with [^18^F]FDG and Na[^18^F]F imaging in mice

**Figure 4:**
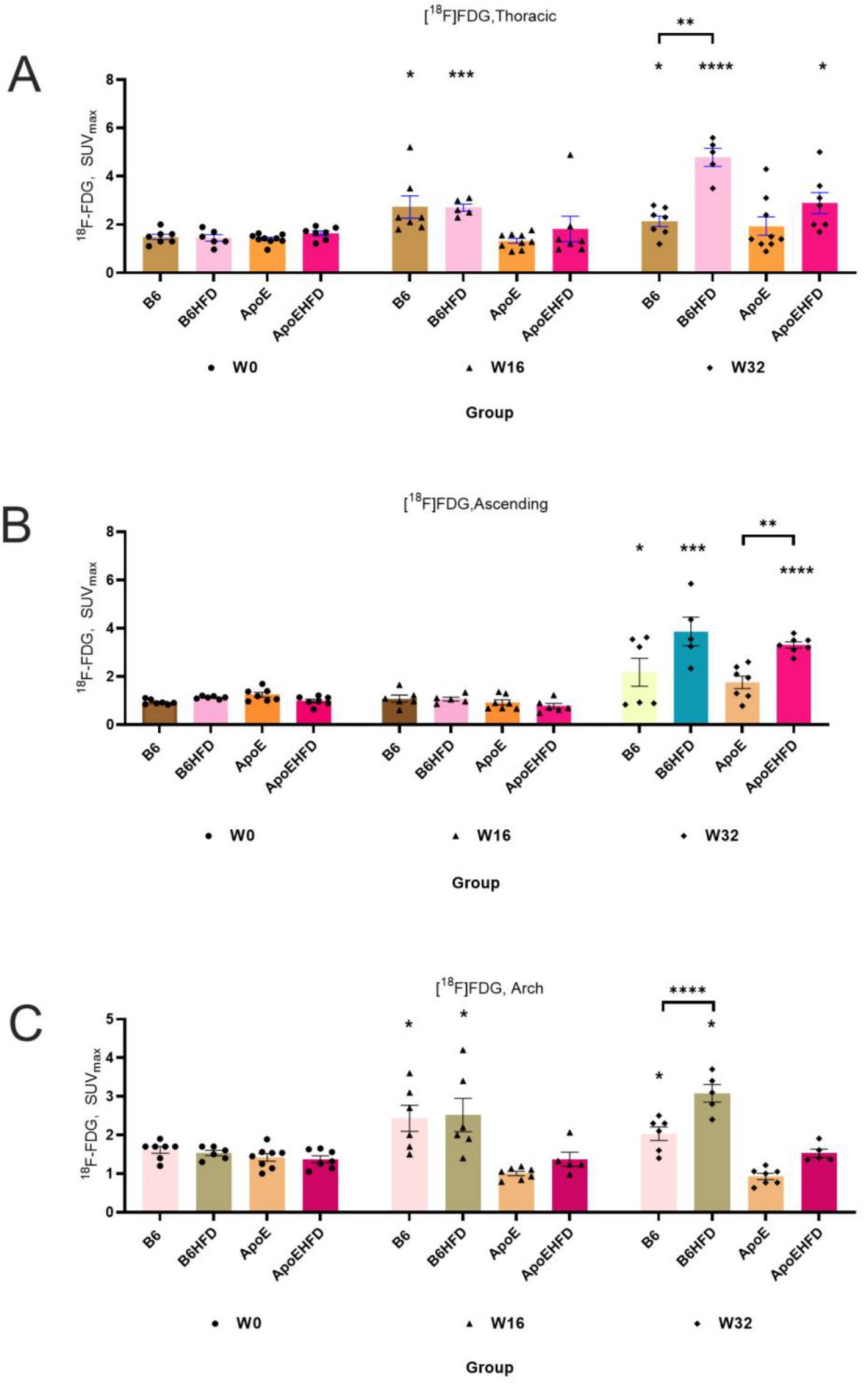
(**A**–**C**) [^18^F]FDG uptakes in three aortic segments in B6, B6HFD, ApoE and ApoEHFD mice at W0, W16 and W32. [^18^F]FDG uptake in the (**A**) Thoracic aorta, (**B**) Ascending aorta and (**C**) Aortic arch at W16 and W32 respectively compared to mean baseline SUV_max_ values. SUV_max_ = Maximum of Standardized uptake values with mean ± std. error of mean, Thoracic = Thoracic aorta, Abdominal = Abdominal aorta, Arch = Aortic arch, W0 = Week 0, W16 = Week16, W32 = Week 32. [^18^F]FDG = 2-deoxy-2-[^18^F]fluoro-D-glucose. Asterisk represents significant differences compared to baseline mean SUV_max_ values. Asterisk on top of a line represents differences between two groups of mice.

Small size of blood vessels, proximity to myocardium and spine (Figs. 1, 2, 3, 4 in Supplementary data) potentially introduces some challenges for PET imaging of small animals such as mice. To analyze the robustness and usefulness of imaging aortic segments other than the abdominal aorta for non-invasive analysis of atherosclerosis in mice, we assessed the [^18^F]FDG and Na[^18^F]F uptake in the aortic arch, where atherosclerosis is usually most pronounced in mice, in the thoracic aorta, where spillover from the spine is a concern for Na[^18^F]F uptake, and the ascending aorta, where quantification might be affected by spillover from the physiological uptake by the myocardium in case of [^18^F]FDG PET. Refer to Figs. 3A–C and 4A–C and Table 2 for thoracic aorta, ascending aorta, aortic arch mean SUV_max_ values and differences in uptakes across mice types and imaging time points.

#### [^18^F]FDG uptake in the thoracic aorta, ascending aorta and the aortic arch

[^18^F]FDG accumulation in the thoracic aorta, the ascending aorta and the aortic arch at W32 resembles the abdominal uptake of [^18^F]FDG in the B6HFD mice group but not in the ApoEHFD group. The uptake in the B6HFD mice thoracic aorta (SUV_max_:4.7 ± 0.3 vs 1.4 ± 0.1, 230.7%, p =  < 0.0001, Fig. 4A), ascending aorta (SUV_max_:3.8 ± 0.5, vs 1.1 ± 0, 240.9%, p = 0.0001, Fig. 4B) and the aortic arch (SUV_max_: 3.0 ± 0.5 vs 1.5 ± 0.2, 100.8%, p =  < 0.0001, Fig. 4C) was observed to be significantly higher compared to B6HFD baseline uptake (Fig. 4C). Significant differences were found between uptakes of B6 & B6HFD mice at W32 in the thoracic aorta (SUV_max_: 2.1 ± 0.2 vs 4.7 ± 0.3, p = 0.0025, Fig. 4A) and the aortic arch (SUV_max_: 2.3 ± 0.2 vs 3.0 ± 0.5, p =  < 0.0001, Fig. 4C) whereas there were significant differences between ApoE and ApoEHFD group in the ascending aorta (SUV_max_: 0.9 ± 0.0 vs 3.2 ± 0.1, p = 0.008, Fig. 4B).

#### Na[^18^F]F uptake in the thoracic aorta

**Figure 5:**
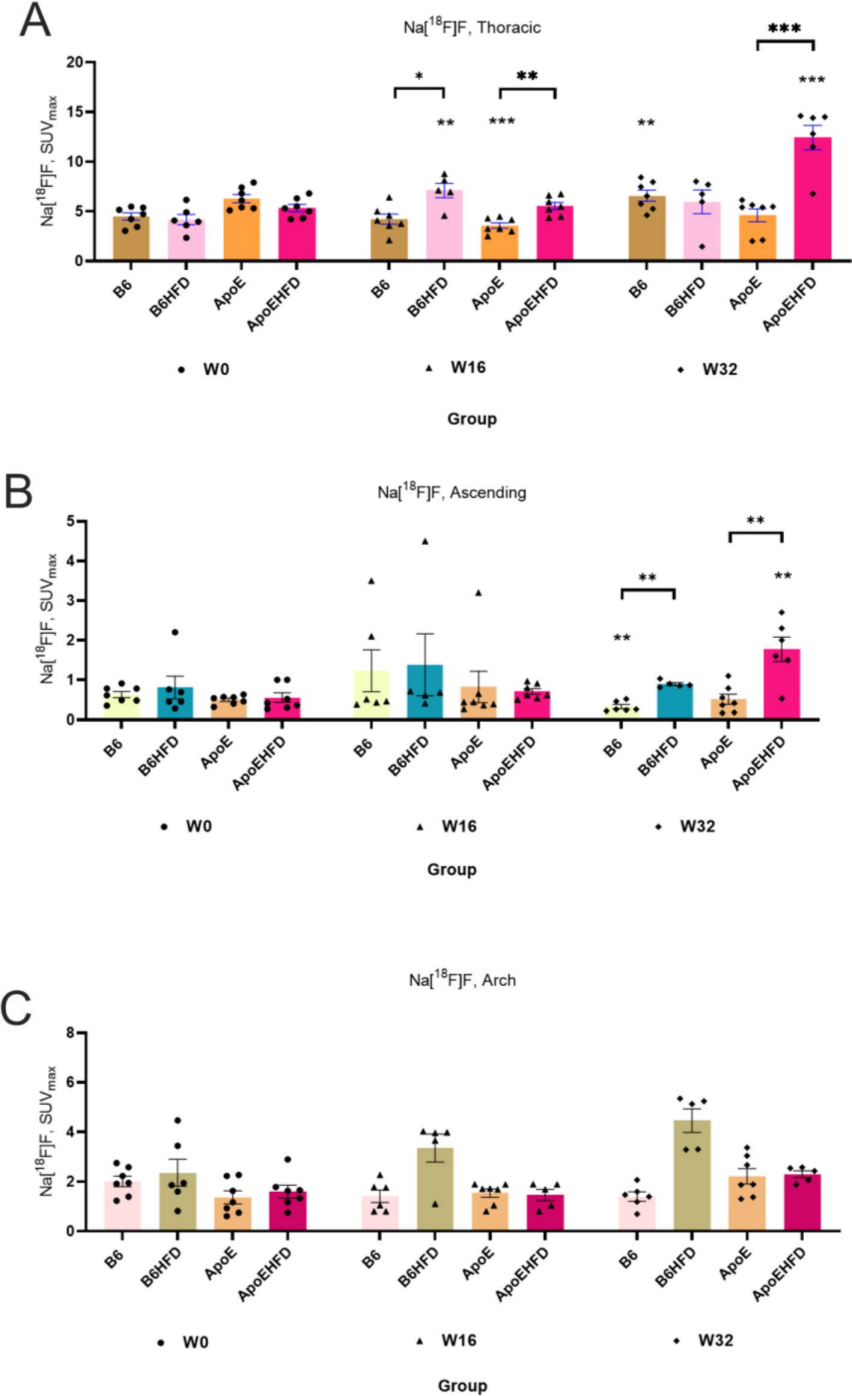
(**A**–**C**) Na[^18^F]F uptakes in three aortic segments in B6, B6HFD, ApoE and ApoEHFD mice at W0, W16 and W32. Na[18F]F uptake in the (**A**) Thoracic aorta, (**B**) Ascending aorta and (**C**) Aortic arch at W16 and W32 respectively compared to mean baseline SUV_max_ values. SUV_max_ = Maximum of Standardized uptake values with mean ± std. error of mean, Thoracic = Thoracic aorta, Abdominal = Abdominal aorta, Arch = Aortic arch, W0 = Week 0, W16 = Week16, W32 = Week 32. [^18^F]NaF = ^18^F-sodium fluoride. Stars represent significant differences compared to baseline mean SUV_max_ values. Asterisk on top of a line represents differences between two groups of mice.

With Na[^18^F]F accumulation, the thoracic aorta corresponds to the uptake of Na[^18^F]F from the abdominal aorta where the highest uptake is observed in the B6HFD (SUV_max_: 7.1 ± 0.7 s 4.1 ± 0.5, 70.2%, p = 0.008, Fig. 5A) group at W16 compared to baseline where significant differences also exists compared to B6 group at W16. At W32, ApoEHFD group showed the highest uptake (SUV_max_: 12.4 ± 1.2 vs 5.3 ± 0.3, 153.7%, p = 0.0001, Fig. 5A) with significant differences between ApoE group (12.4 ± 1.2 vs 4.6 ± 0.6, p = 0.01, Fig. 5A).

Details of *p* values resulting from parametric unpaired t test analyses between [^18^F]FDG & Na[^18^F]F uptakes in different mice groups and aortic segments are reported in Table 3. Spaghetti plots representation of data from Figs. 3, 4 and 5 are illustrated in Supplementary Figs. 5, 6 and 7.

**Table 3:** Comparison of diet induced changes in the two mouse strains for [^18^F]FDG & Na[^18^F]F uptakes in the different aortic segments.

	B6 vs B6HFD	ApoE vs ApoEHFD	B6 vs B6HFD	ApoE vs ApoEHFD
	W16	W16	W32	W32
[^18^F]FDG				
Thoracic	ns	ns	0.0025(**)	ns
Ascending	ns	ns	ns	0.008 (**)
Abdominal	0.015(*)	ns	0.006(**)	ns
Arch	ns	ns	< 0.0001(****)	ns
[^18^F]NaF				
Thoracic	ns	0.01 (*)	ns	0.001 (***)
Ascending	ns	0.01 (*)	0.006(**)	0.008 (**)
Abdominal	ns	0.02 (*)	0.006(**)	0.02(*)
Arch	ns	0.02 (*)	ns	ns

Shown are *p* values resulting from parametric unpaired t test analyses.

To assess potential differences in BAT and muscle uptake of [^18^F]FDG SUV_mean_ between diets and mouse strains, TBR_max_ values (Aorta to BAT & Aorta to Muscle) were calculated. Results are illustrated in Supplementary Figs. 8A and 9A as BAT and Muscle SUV_mean_ and TBR_max_ values in Supplementary Figs. 8B and 9B, respectively.

### Histological demonstration of calcification and Ex vivo quantification of Na[^18^F]F accumulation

**Figure 6:**
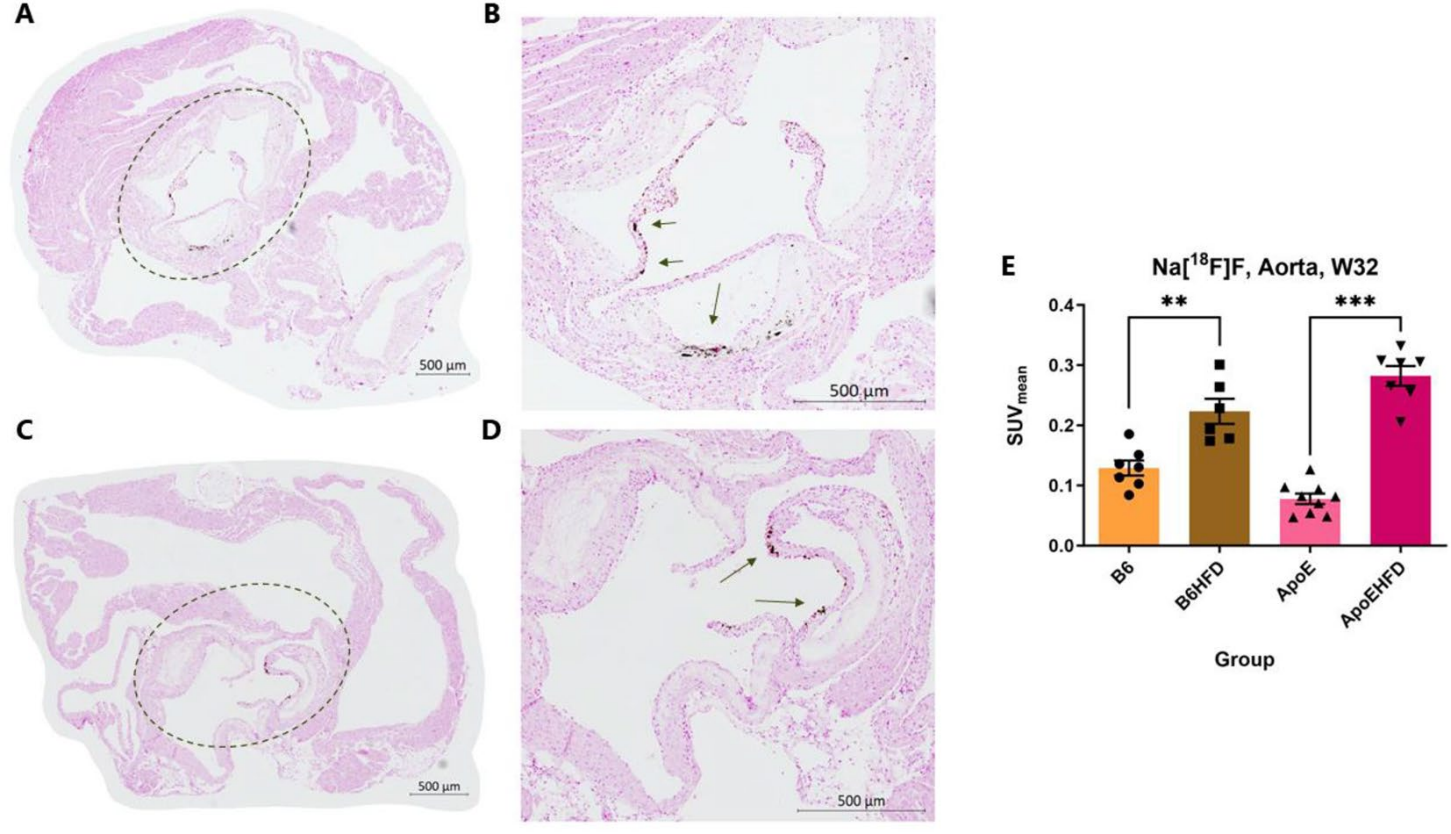
Ex vivo visualization of calcification by Von Kossa stain and quantification of Na[^18^F]F accumulation in mice aortas by gamma counting. (**A**–**D**) Representative images of aortic roots showing Von Kossa staining in ApoEHFD mice where areas marked by dotted circles (in (**A**) and (**C**)) are magnified (in (**B**) and (**D**)) and staining is marked by arrows. (**E**) Gamma counts in terms of Na[18F]F SUVmean values resulting from dissected whole aortas 2 from B6 group (N = 7), B6HFD group (N = 6), ApoE group (N = 9) and ApoEHFD group (N = 7). Data presented as mean of standardized uptake values (SUV_mean_) ± std. error of mean, Na[^18^F]F = ^18^F-sodium fluoride. Asterisks represent level of significant differences between groups.

Presence of mineralization in the aortas of mice was confirmed with Von Kossa staining where the staining was observed lining the lesion areas, marked by circle and arrows in Fig. 6A to D. Excised whole aortas were also gamma counted to confirm and quantify accumulation of Na[^18^F]F. Highest counts in terms of average of SUV_mean_ values were observed in ApoEHFD group (0.282 ± 0), followed by B6HFD group (0.223 ± 0), ApoE group (0.077 ± 0) and B6 group (0.129 ± 0). Significant differences were observed between ApoEHFD group and ApoE group (p = 0.0002), B6HFD group and B6 group (p = 0.004) (Fig. 6E).

## Discussion

We have previously demonstrated [^18^F]FDG imaging of ApoE KO mice on NCD and HFD over 32 weeks with increase in SUV_mean_ values over imaging time-points at Week 8, 16, 24 and 32. We further demonstrated a strong correlation between PET and gamma counting with [^18^F]FDG. In the current study, we add Na[^18^F]F to measure and visualize active calcifications along with [^18^F]FDG and the commonly used experimental mouse strain, the B6 mice (on both NCD & HFD).

Overall, we demonstrate non-invasive imaging of inflammation and calcification in specific aortic branches of mice. Our data suggests that for both tracers, abdominal aorta is the preferred segment of image readout. While looking at uptakes of tracers by specific mice strains, B6HFD showed maximum increase in [^18^F]FDG uptake in the thoracic aorta and the aortic arch whereas ApoEHFD mice showed maximum increase in [^18^F]FDG uptake in the ascending aorta, abdominal aorta. In fact, although the relative difference between HFD and NCD are similar but as the inflammation in ApoE mice also increases without HFD the use of B6, where the baseline level is stable, may even represent an advantage over ApoE mice. The data therefore underscores that the proper, strain specific controls should always be used. For Na[^18^F]F, the abdominal and thoracic aorta seems the best regions to use but due to the close proximity of the spine, spill over cannot be ruled out for Na[^18^F]F in the thoracic region. Accordingly, the abdominal aorta seems to be the primary choice for both the tracers. For microcalcification, which is a later phenomenon than inflammation and that can be visualized with Na[^18^F]F, Maximum increase in Na[^18^F]F uptake is observed in ApoEHFD mice.Here, it seems necessary to use ApoE mice as the changes are too subtle in B6 mice^24,25^. The in vivo PET/CT results are further confirmed by ex vivo gamma counting of Na[^18^F]F accumulation where the results show a similar trend.

There are several points to consider when using [^18^F]FDG in mice for detecting atherosclerosis. Mice are known to have brown adipose tissue (BAT) in the neck region which is prone to accumulate [^18^F]FDG. Proximity of brown fat as well as myocardial tissue to arterial segments poses a challenge in vascular imaging^26,27^. Accumulation in brown fat was minimized by assuring that the mice were kept warm at all times following tracer injection and during scans. Despite these precautions, we still observed some differences in BAT [^18^F]FDG uptake between the groups, and awareness should be given to the potential impact this might have on the results. A previous study with [^18^F]FDG imaging in ApoE knock-out mice reported on brown fat as a confounding factor^28^. Extended periods on high fat diet might introduce obesity along with atherosclerosis in both the mice strains and this might have an effect on the accumulation and quantification of [^18^F]FDG. Myocardial [^18^F]FDG uptake was also observed in the current study but did not seem to affect the quantification in the ascending aorta, since the results obtained here were in line with or lower than the other segments analyzed. In addition to the differential uptake of [^18^F]FDG in BAT, we observed that diet and mouse strain also affected the tracer accumulation in muscle, where all other groups than the B6 showed an increase in [^18^F]FDG uptake at Week 16 and 32.

While Na[^18^F]F is not as prone as [^18^F]FDG to accumulate in all metabolically active cells, Na[^18^F]F is readily taken up by bones. Therefore, the proximity of the aorta to the spine and its potential spill-over should also be considered. Refer to Supplementary Fig. S4 for a representative example of Na[^18^F]F uptake observed in bones. We did observe a higher uptake in the thoracic and abdominal aorta compared to the arch and ascending aorta, but the results from all 4 regions were in agreement with significantly higher uptake at W32 in the ApoEHFD group compared to the other groups and when compared to baseline. The quantification of Na[^18^F]F accumulation via PET follows a similar trend shown by ex vivo gamma counting. The slight discrepancy observed are most likely to be the spill-over effects and from the fact that ex vivo quantification is not segmented as it has been with PET quantification.

At W32, we found CT detectable calcification in line with the stable and very advanced stage of atherosclerosis and this was further confirmed by evidence of mineralization in the aortas by Von Kossa staining. These macro-calcifications did not seem to absorb Na[^18^F]F, possibly due to it being ‘non-active’ or large in size. Studies suggest that micro-calcifications are less than 50 µm in size and are undetectable on a CT scan as compared to macro-calcifications^29,30^.This puts an emphasis on Na[^18^F]F as a PET tracer used for detection of only active or micro-calcifications. Irkle et al. demonstrated a high correlation between Na[^18^F]F and Alizarin red calcification staining in human atherosclerotic tissue and our study confirms this finding^29^. A study with [^18^F]FDG & Na[^18^F]F PET/CT in a rabbit model of atherosclerosis shows correlation with PET/CT quantification and autoradiography in control and atherosclerotic rabbits^31^. Furthermore, our study demonstrates that Na[^18^F]F can detect micro calcifications not seen on a CT scan. The extensive head-to-head comparison presented in this study suggests Na[^18^F]F accumulate in active calcifications providing a complementary tool to [^18^F]FDG imaging. Inflammation and microcalcification represent two early steps in development of atherosclerosis and thus helps understand better how the disease develop.

The physical properties in terms of half-life and positron range are in the same range for both tracers due to both being ^18^F-labelled^32^. Positron range of 0.6 mm by ^18^F is amongst the shortest in commonly used radioisotopes for imaging purposes^32,33^. Despite having this advantage, spatial resolution of PET when imaging small animals such as in mice is limited^34^. The combined resolution is with state-of-the-art preclinical PET/CT scanners in the range of 1.2 mm – for comparison, the diameter of the mouse aorta is 1.0 to 1.2 mm in B6 mice^35–38^. In the current study we demonstrate that this is sufficient for relevant readout in the mouse aorta for following development of atherosclerosis. However, as mentioned above, spill-over from other areas as well as unavoidable partial volume effects must be taken into account and represent limitations of the method. It may, however, be possible to at least in part correct for the partial volume effect as recently shown for Na[^18^F]F imaging in mice^39^.

Addition of a contrast agent in our study was a great benefit in identifying small anatomical structures including the aorta in mice. This aided a more precise ROI placement, consequently resulting in accurate observations. In the future, AI (Artificial Intelligence) based segmentation and interpretation could be used to increase the efficiency, accuracy and make it devoid of human errors and bias^40,41^.

Taken together, our study demonstrates a robust, reliable method in segmenting and quantification in different aortic segments of mice.

## Conclusion

The uptake of both [^18^F]FDG and Na[^18^F]F increased as the disease progressed over time and accumulation in atherosclerotic groups were most pronounced at the very advanced stage (W32). The abdominal aorta provides a robust measure across mouse strain and diet. Therefore, it seems to be the preferred region for image readout in atherosclerotic mouse studies. Awareness should be given to the variable uptake of ^18^F]FDG in brown adipose tissue and muscle between mouse strains and diets in cardiovascular imaging.

For [^18^F]FDG -PET, both B6 and ApoE mice provide valuable information and either mouse strain could be used in preclinical cardiovascular studies, whereas regarding Na[^18^F]F uptake, a significantly altered uptake was observed only in ApoE mice and therefore these mice should be preferred.

### Supplementary Information

Supplementary Figures.

## Data Availability

The datasets used and analyzed during the current study are available from the corresponding author on a reasonable request.
