# Supplementary material for: Longitudinal imaging of murine atherosclerosis with 2-deoxy-2-[18F]fluoro-D-glucose and [18F]-sodium fluoride in genetically modified Apolipoprotein E knock-out and wild type mice
Source: Sci Rep. 2023 Dec 27;13:22983. doi: 10.1038/s41598-023-49585-1 (PMC10752895; doi:10.1038/s41598-023-49585-1)
Supplement: Supplementary file 1 — Supplementary Figures. [file 41598_2023_49585_MOESM1_ESM.pdf]

Supplementary data

Supplementary Figure 1

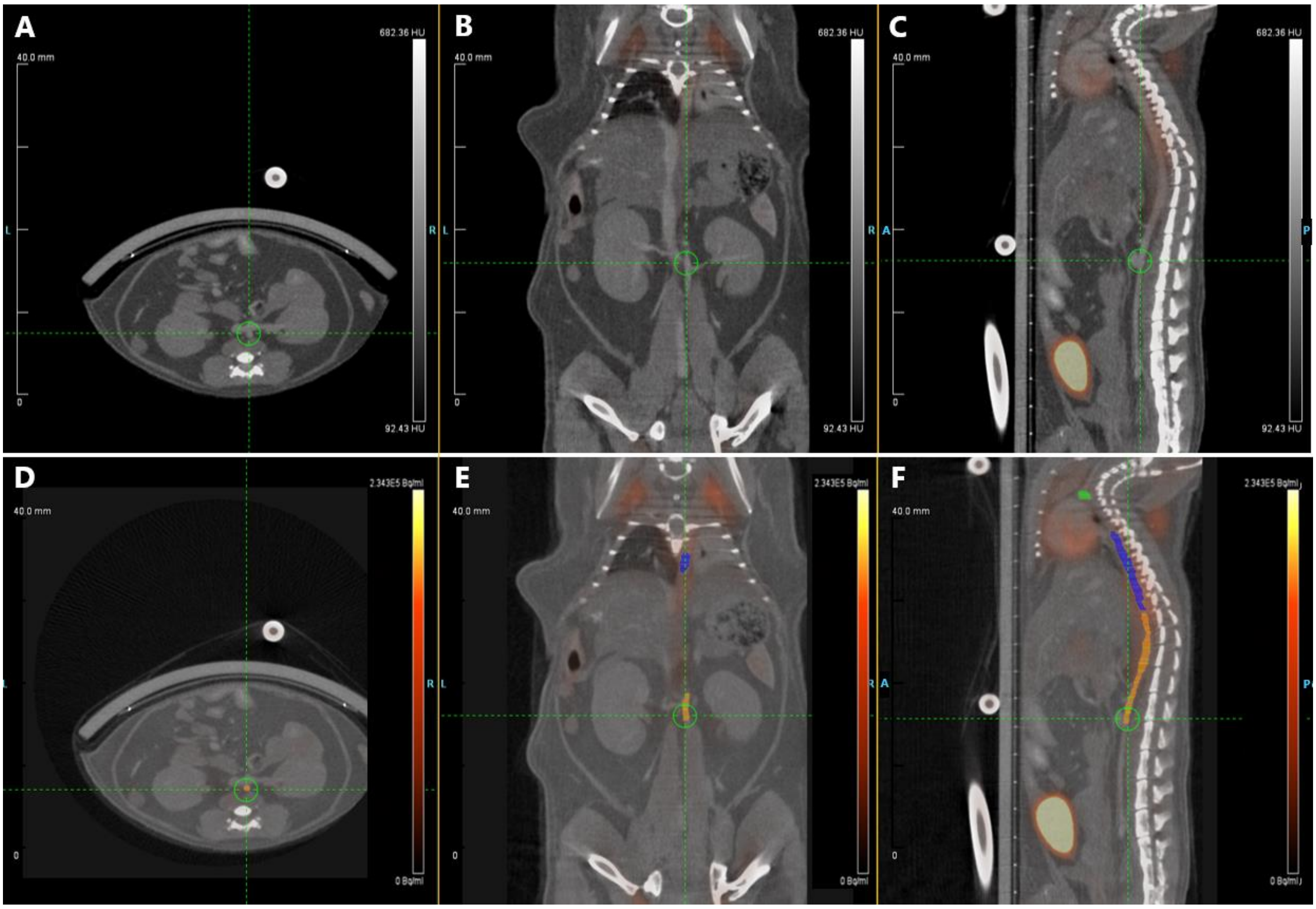

Supplementary Figure 1. Segmentation of aorta A. Representative [^{18}F]FDG PET/CT scan of an ApoEHFD mouse at W32 with axial (left), coronal (middle) and sagittal (right) views with the pointer (in green) at the renal artery bifurcation. **B.** ROIs were drawn starting from this point on every fifth axial slice cranially to the aortic arch. **C.** Complete aorta is visible in the sagittal view without ROIs and highlights. **D & E.** ROIs were drawn and connected to calculate volumes and segment the aorta. **F.** Aortic segments highlighted as abdominal aorta (in orange), thoracic aorta (in blue) and aortic arch (in green). L=Left, R= Right, A= Anterior, P= Posterior.

Supplementary Figure 2

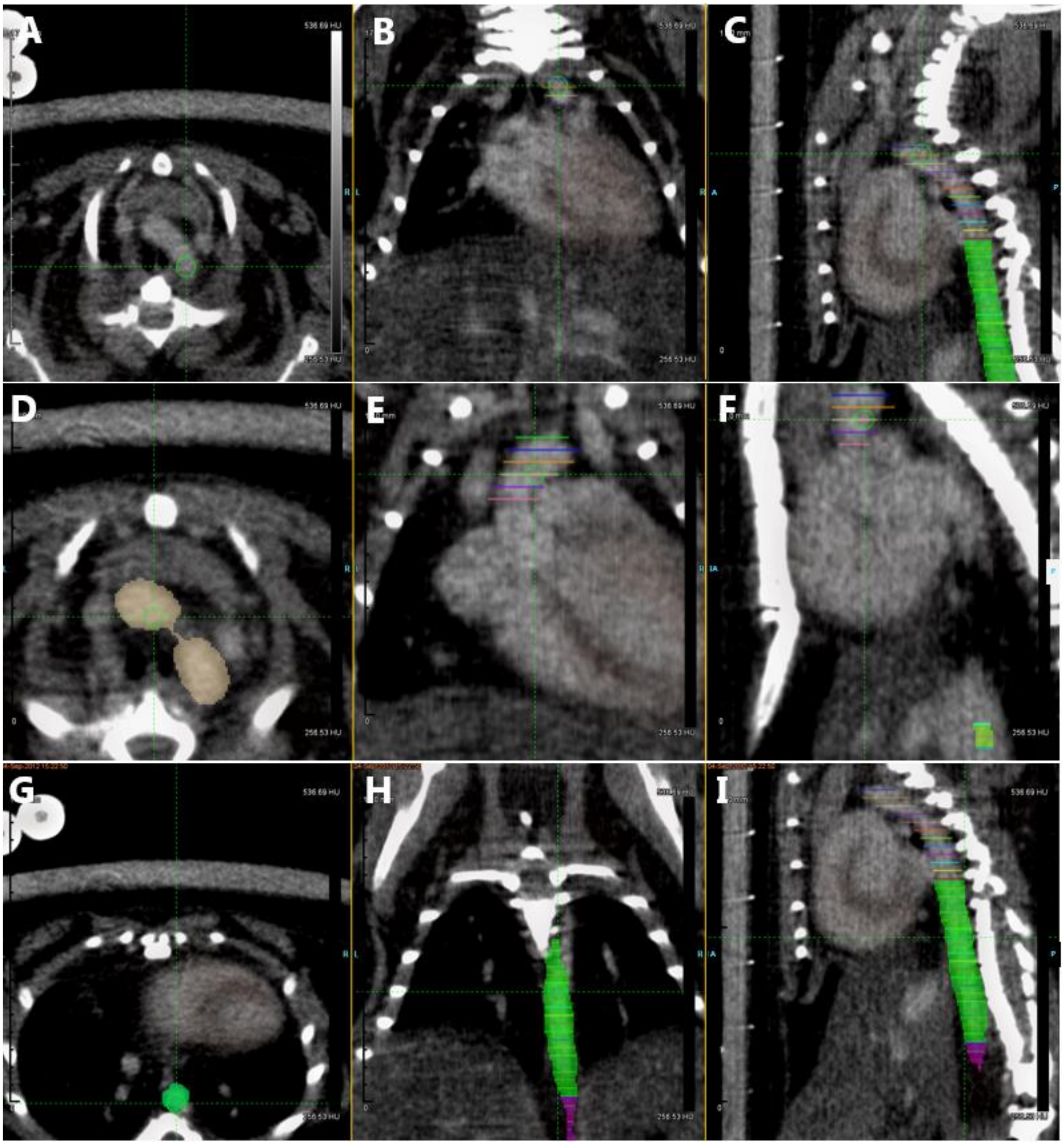

Supplementary Figure 2. Representative PET/CT scan from an ApoEHFD mouse at W32. A, B & C. Pointer placed on the aortic arch showing the arch in three different views. **D, E & F**. ROIs drawn on the aortic arch **(D)**, ascending aorta **(E)** and a highlighted (in green) thoracic aorta **(F)**. **G, H & I**. Pointer placed on thoracic aorta showing it in three views and its proximity to the spine and heart. Left to right -Axial, Coronal, Sagittal views. L=Left, R= Right, A= Anterior, P= Posterior.

Supplementary Figure 3

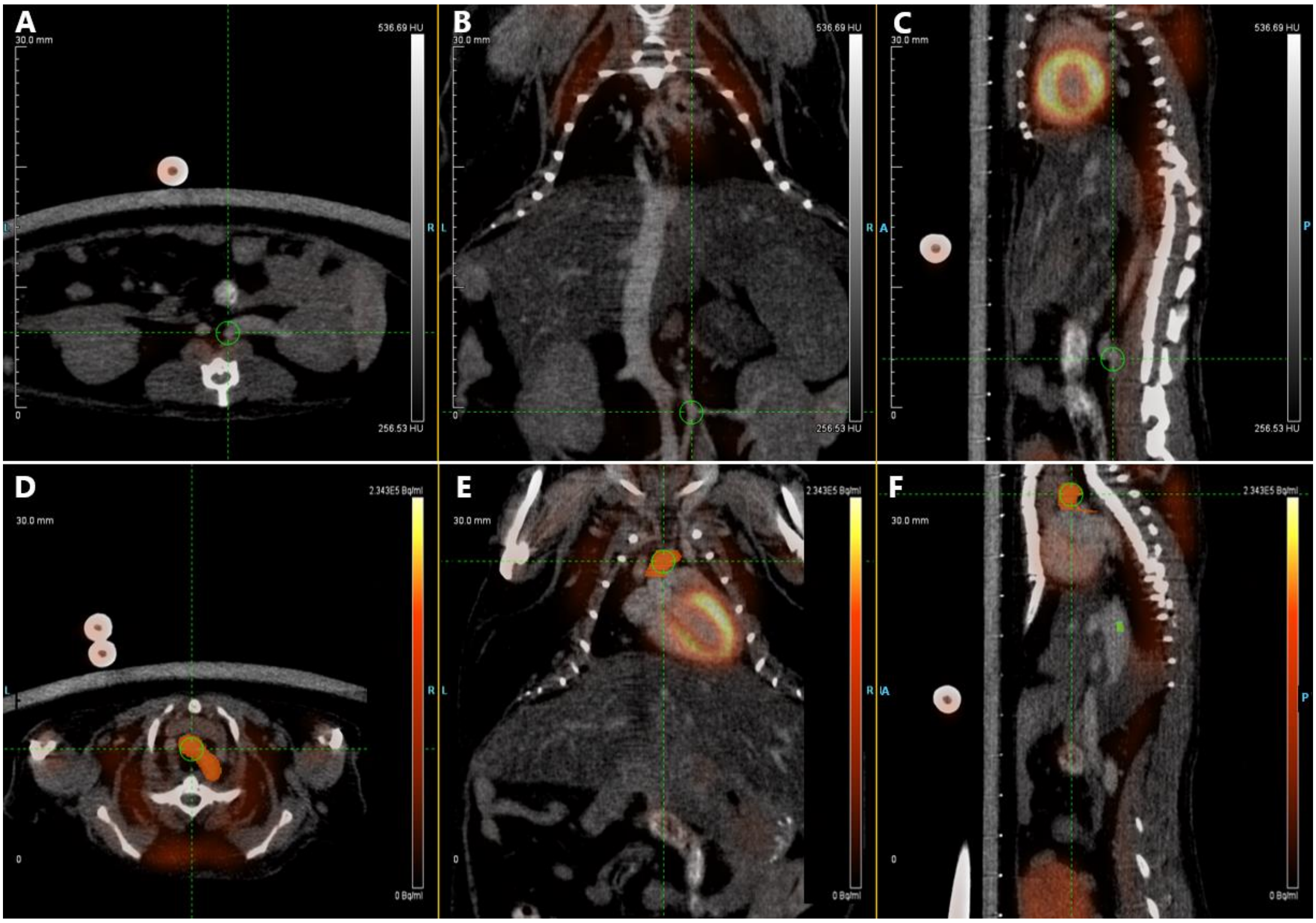

Supplementary Figure 3. Myocardial [^{18}F]FDG uptake. A & B. Pointer placed on renal bifurcation in order to display uptake in the heart visible in saggital view **(C)**. **D, E & F**. Pointer placed on the aortic arch showing myocardial uptake prominantly in coronal view. Left to right -Axial, Coronal, Sagittal views. L=Left, R= Right, A= Anterior, P= Posterior.

Supplementary Figure 4

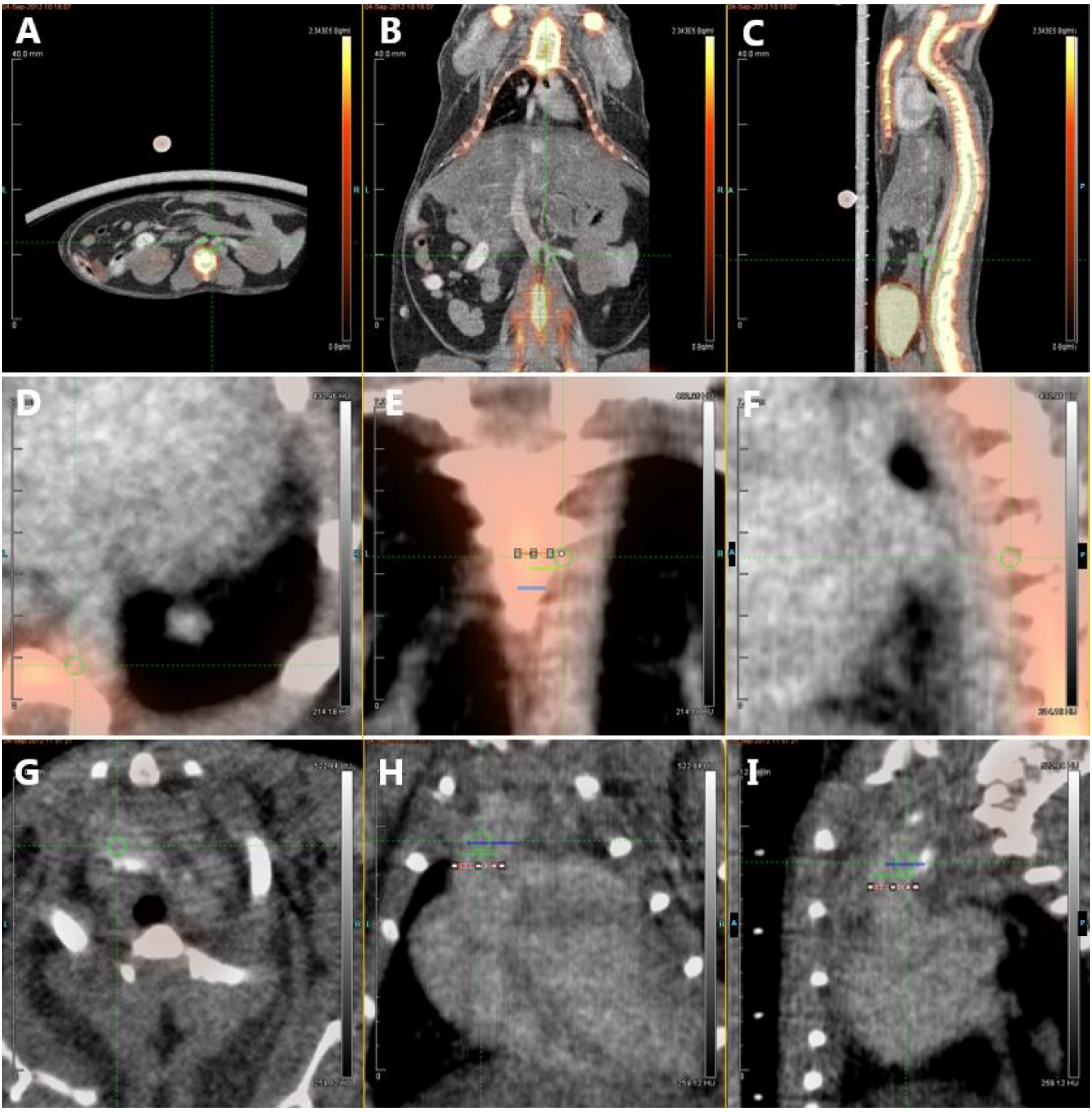

Supplementary Figure 4. Na[^{18}F]F uptake observed in bones. A to F. Na[^{18}F]F uptake by skeleton and the proximity of aorta to the spine in different views. **G, H & I**. CT visible calcification observed close to the green colored pointer in the aortic arch **(G)** and ascending aorta **(H)** of a ApoEHFD mouse most prominently visible on axial **(L)** and sagittal views **(R)**. Left to right -Axial, Coronal, Sagittal views. L=Left, R= Right, A= Anterior, P= Posterior.

Supplementary Figure 5

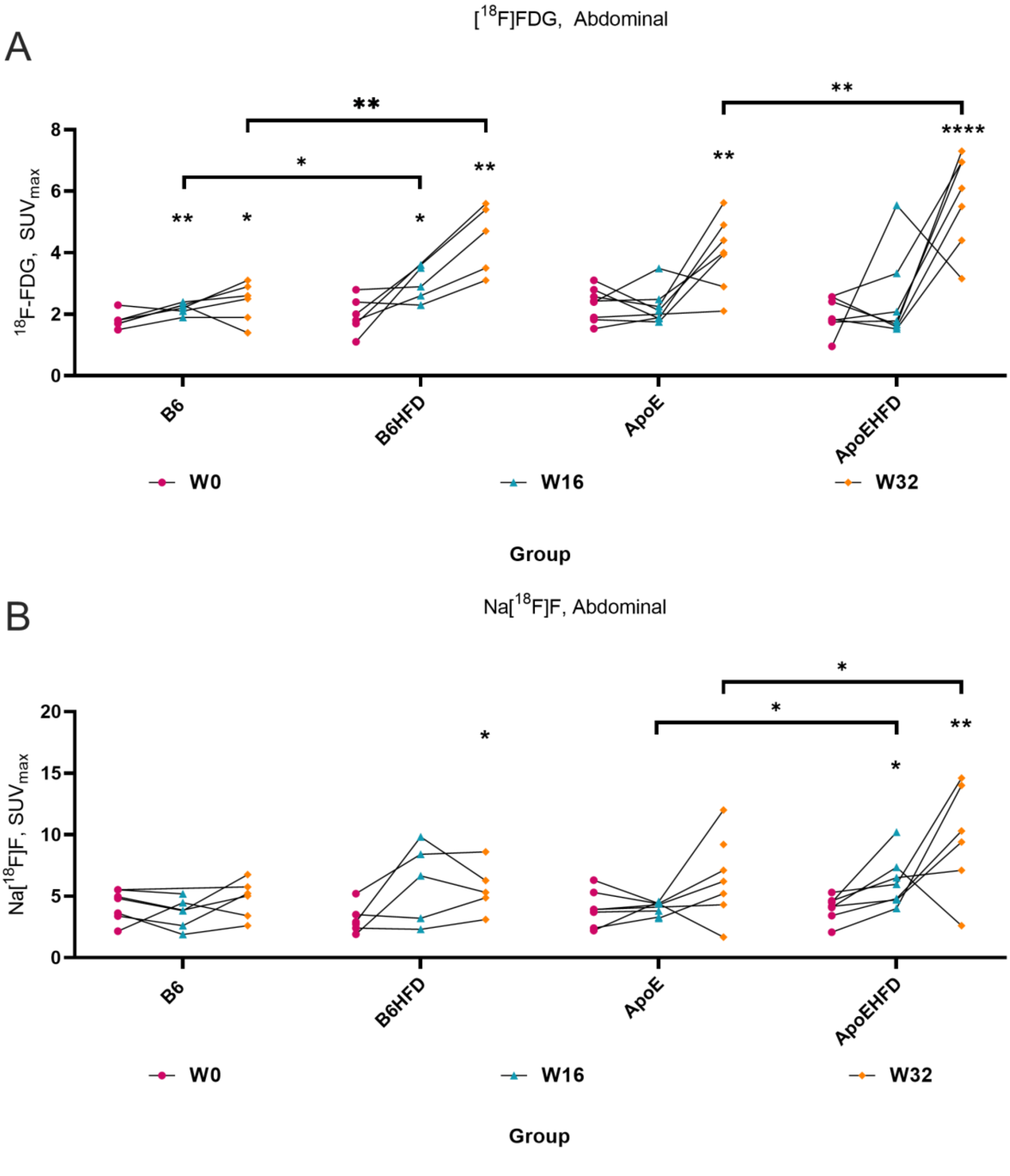

Supplementary Figure 5. Spaghetti plot representation of [^{18}F]FDG and Na[^{18}F]F uptakes by PET/CT imaging of atherosclerosis in abdominal aorta in Figure 5.A & Figure 5.B respectively. Highest uptakes and increases in mean SUVmax values of 18[F]FDG and Na[^{18}F]F were observed in ApoEHFD mice in the abdominal aorta at W32. SUV_{max}= Maximum of Standardized uptake values with mean ± std. error of mean, Refer to Table 2 for %increases in mean SUV_{max} and p values. [^{18}F]FDG= 2-deoxy-2-[^{18}F]fluoro-D-glucose. Na[^{18}F]F= ^{18}F-sodium fluoride. Asterisks represent significant differences compared to baseline mean SUV_{max} values from the respective group. Asterisks with a line represent significant differences within high fat diet group and chow diet group.

Supplementary Figure 6

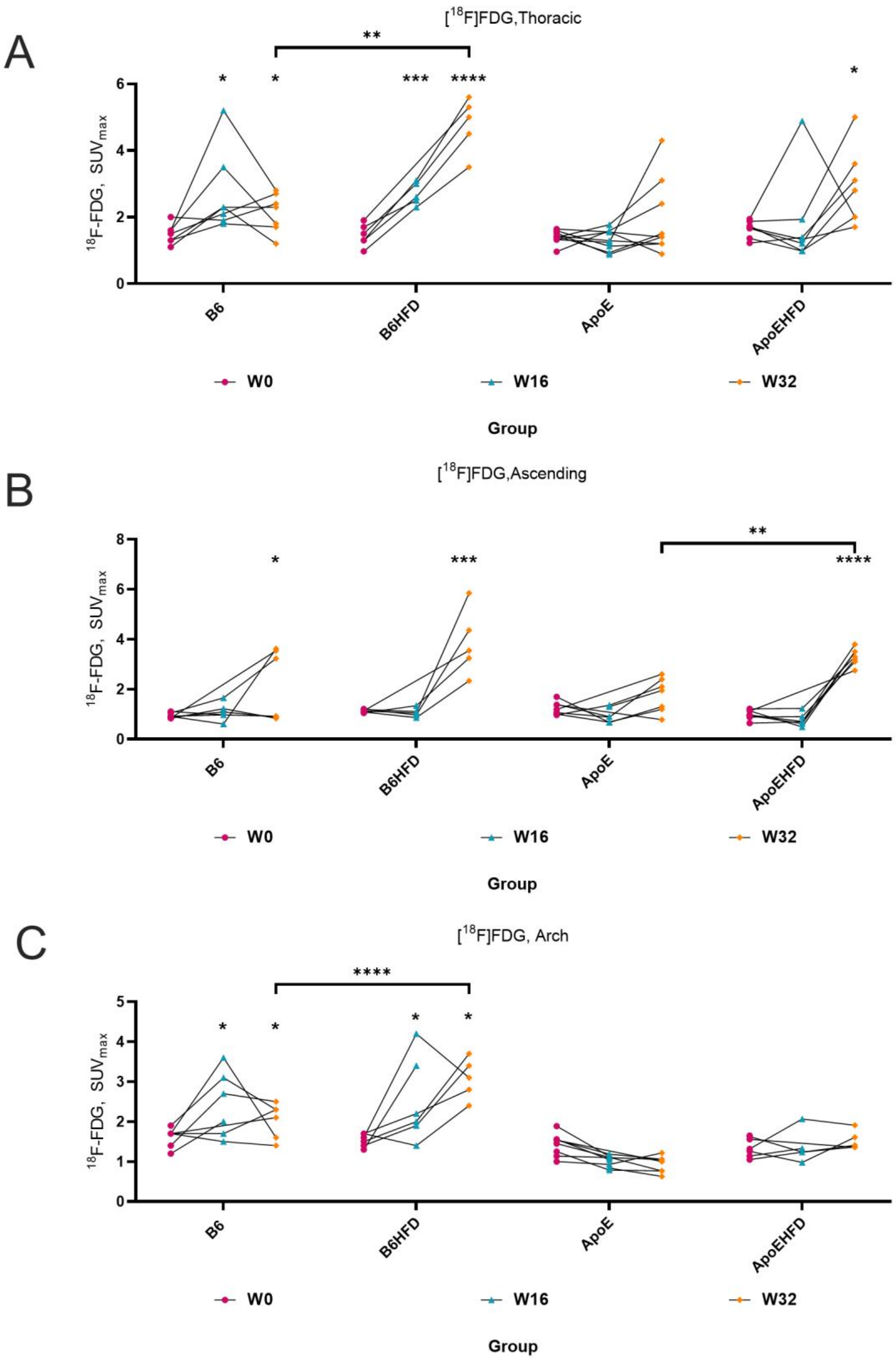

Supplementary Figure 6.A, B & C. [^{18}F]FDG uptakes in three aortic segments in B6, B6HFD, ApoE and ApoEHFD mice at W0, W16 and W32.

[^{18}F]FDG uptake in the **A.** thoracic aorta, **B.** ascending aorta and **C.** aortic arch at W16 and W32 respectively compared to mean baseline SUV_{max} values. SUV_{max}= Maximum of Standardized uptake values with mean ± std. error of mean, Thoracic= Thoracic aorta , Abdominal= Abdominal aorta, Arch= Aortic arch , W0= Week 0, W16= Week16, W32= Week 32. [^{18}F]FDG= 2-deoxy-2-[^{18}F]fluoro-D-glucose. Asterisks represent significant differences compared to baseline mean SUV_{max} values. Asterisks with a line represent significant differences within high fat diet group and chow diet group.

Supplementary Figure 7

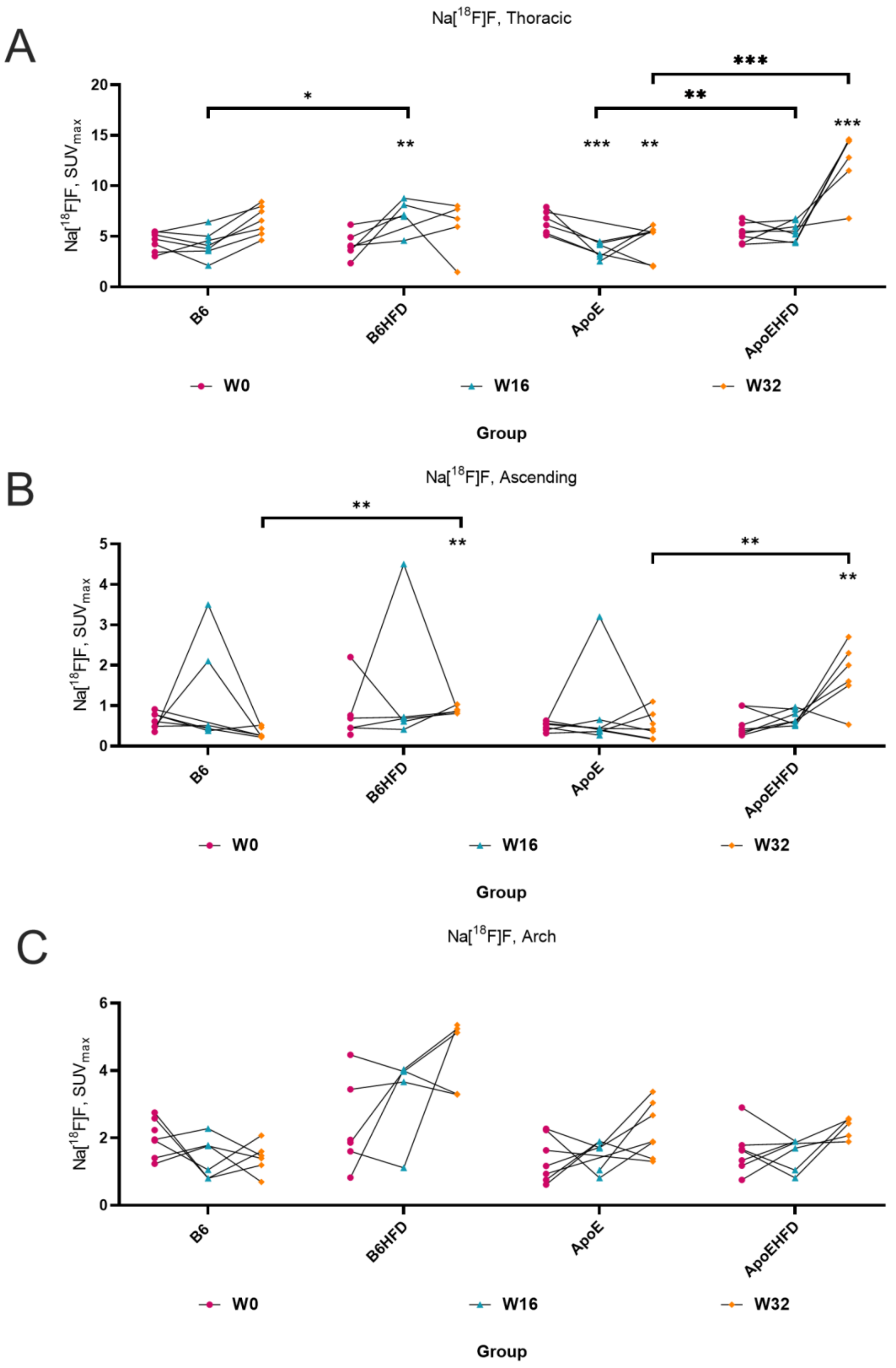

Supplementary Figure 7.A, B & C. Na[^{18}F]F uptakes in three aortic segments in B6, B6HFD, ApoE and ApoEHFD mice at W0, W16 and W32.

Na[^{18}F]F uptake in the **A.** thoracic aorta, **B.** ascending aorta and **C.** aortic arch at W16 and W32 respectively compared to mean baseline SUV_{max} values. SUV_{max}= Maximum of Standardized uptake values with mean ± std. error of mean, Thoracic= Thoracic aorta , Abdominal= Abdominal aorta, Arch= Aortic arch , W0= Week 0, W16= Week16, W32= Week 32. Na[^{18}F]F= ^{18}F-sodium fluoride. Asterisks represent significant differences compared to baseline mean SUV_{max} values. Asterisks with a line represent significant differences within high fat diet group and chow diet group.

Supplementary Figure 8

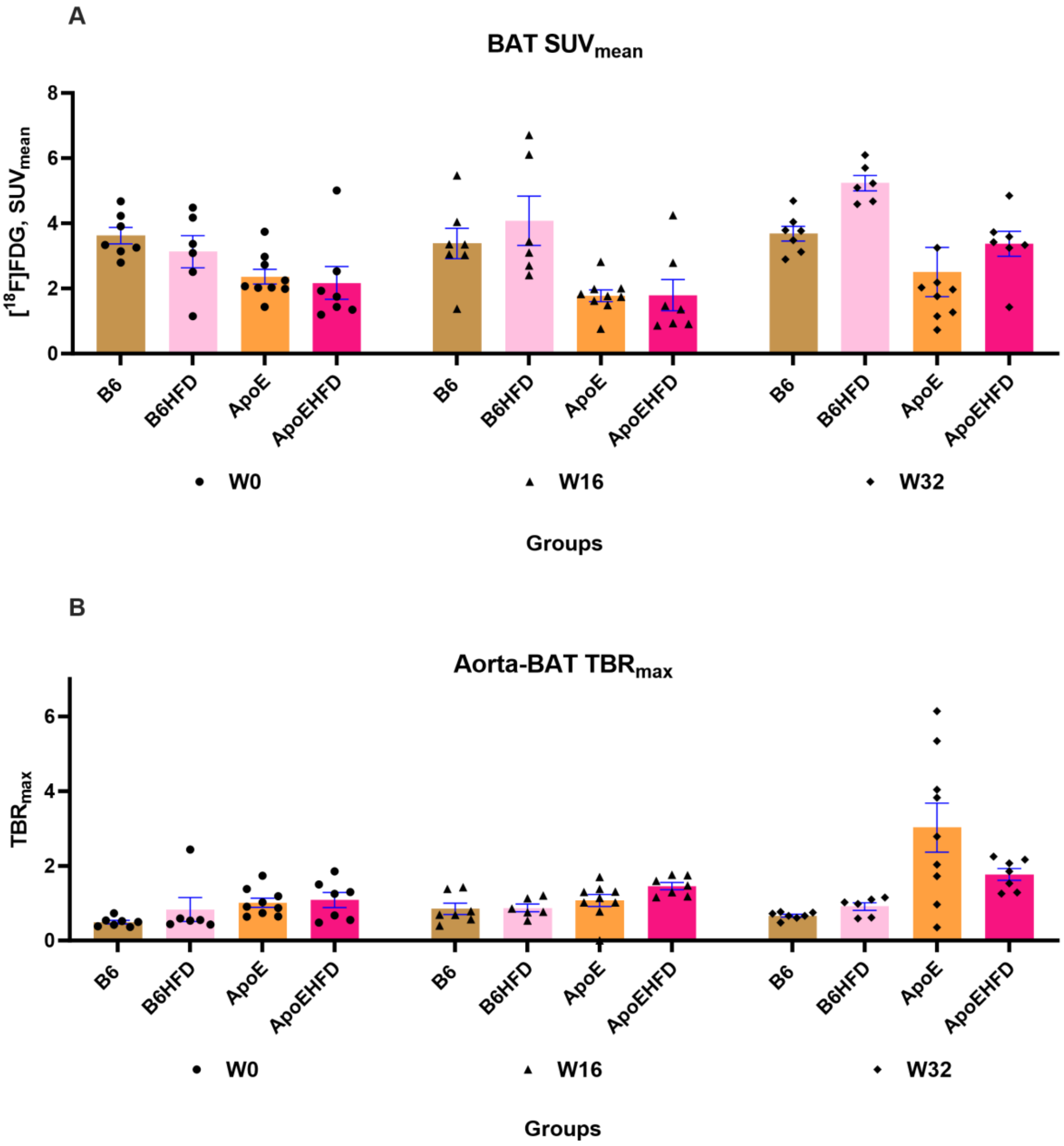

Supplementary Figure 8.A & B. $[^{18}F]$FDF SUV_{mean} values from BAT across all mice groups and timepoints and resulting TBR_{max} values. A.$[^{18}F]$FDG uptake in the brown adipose tissue fat (BAT) in SUV_{mean} values from B6, B6HFD, ApoE and ApoEHFD mice groups at W0, W16 and W32. **B.** Maximum of abdominal aorta to BATF (Tissue to Background ratio, TBRmax) from B6, B6HFD, ApoE and ApoEHFD mice groups at W0, W16 and W32.

Supplementary Figure 9

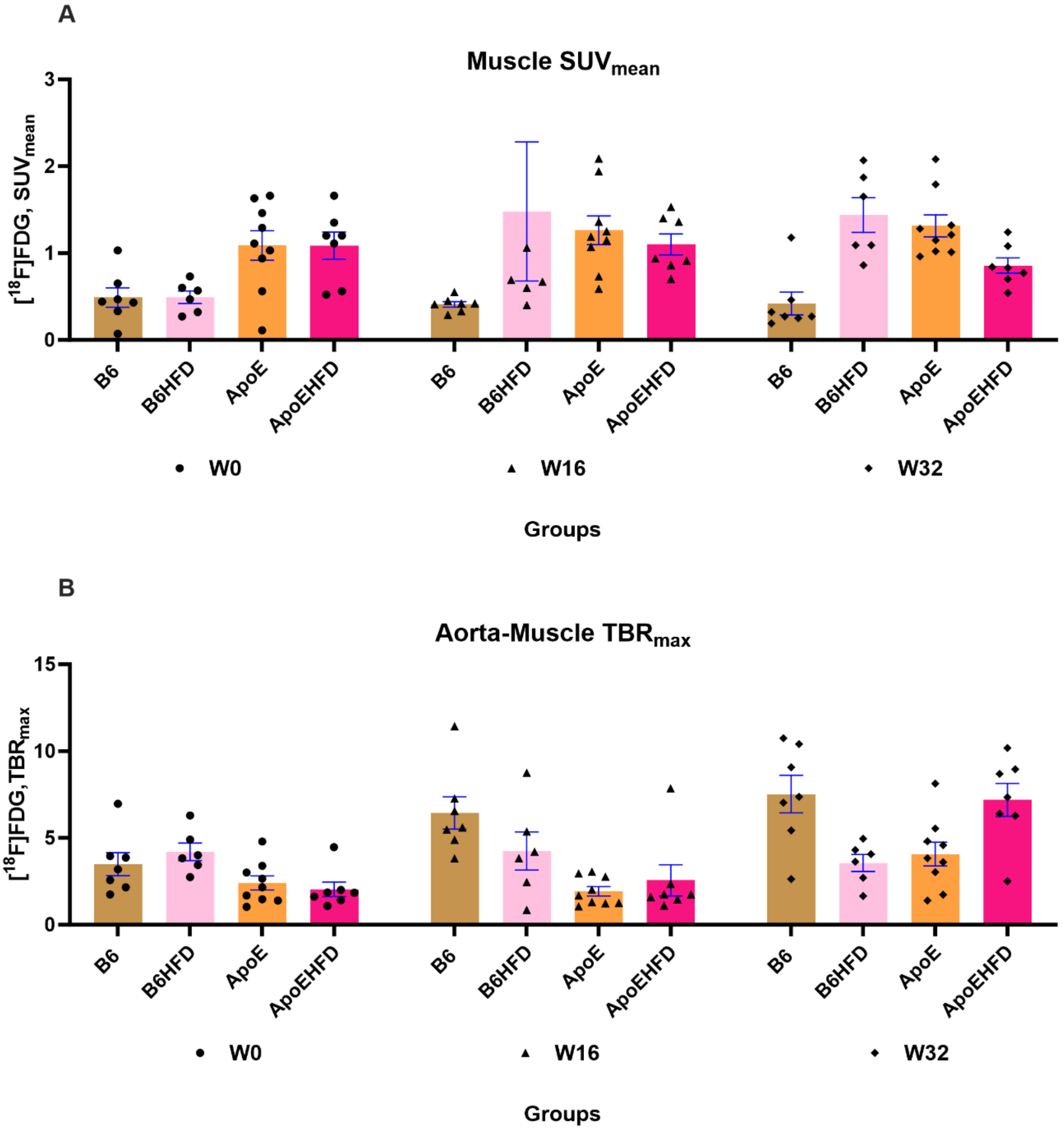

Supplementary Figure 9.A & B. [^{18}F]FDF SUV$_{mean}$ values from muscle across all mice groups and timepoints and resulting TBR$_{max}$ values. A.[^{18}F]FDG uptake in the quadricep muscle in SUV$_{mean}$ values from B6, B6HFD, ApoE and ApoEHFD mice groups at W0, W16 and W32. **B.** Maximum of abdominal aorta to muscle (Tissue to Background ratio, TBRmax) from B6, B6HFD, ApoE and ApoEHFD mice groups at W0, W16 and W32.
